# Self-Regulation of Amygdala Activation Using Real-Time fMRI Neurofeedback

**DOI:** 10.1371/journal.pone.0024522

**Published:** 2011-09-08

**Authors:** Vadim Zotev, Frank Krueger, Raquel Phillips, Ruben P. Alvarez, W. Kyle Simmons, Patrick Bellgowan, Wayne C. Drevets, Jerzy Bodurka

**Affiliations:** 1 Laureate Institute for Brain Research, Tulsa, Oklahoma, United States of America; 2 Department of Molecular Neuroscience, George Mason University, Fairfax, Virginia, United States of America; 3 Department of Psychology, George Mason University, Fairfax, Virginia, United States of America; University of Muenster, Germany

## Abstract

Real-time functional magnetic resonance imaging (rtfMRI) with neurofeedback allows investigation of human brain neuroplastic changes that arise as subjects learn to modulate neurophysiological function using real-time feedback regarding their own hemodynamic responses to stimuli. We investigated the feasibility of training healthy humans to self-regulate the hemodynamic activity of the amygdala, which plays major roles in emotional processing. Participants in the experimental group were provided with ongoing information about the blood oxygen level dependent (BOLD) activity in the left amygdala (LA) and were instructed to raise the BOLD rtfMRI signal by contemplating positive autobiographical memories. A control group was assigned the same task but was instead provided with sham feedback from the left horizontal segment of the intraparietal sulcus (HIPS) region. In the LA, we found a significant BOLD signal increase due to rtfMRI neurofeedback training in the experimental group versus the control group. This effect persisted during the Transfer run without neurofeedback. For the individual subjects in the experimental group the training effect on the LA BOLD activity correlated inversely with scores on the Difficulty Identifying Feelings subscale of the Toronto Alexithymia Scale. The whole brain data analysis revealed significant differences for Happy Memories versus Rest condition between the experimental and control groups. Functional connectivity analysis of the amygdala network revealed significant widespread correlations in a fronto-temporo-limbic network. Additionally, we identified six regions — right medial frontal polar cortex, bilateral dorsomedial prefrontal cortex, left anterior cingulate cortex, and bilateral superior frontal gyrus — where the functional connectivity with the LA increased significantly across the rtfMRI neurofeedback runs and the Transfer run. The findings demonstrate that healthy subjects can learn to regulate their amygdala activation using rtfMRI neurofeedback, suggesting possible applications of rtfMRI neurofeedback training in the treatment of patients with neuropsychiatric disorders.

## Introduction

Real-time functional magnetic resonance imaging (rtfMRI), in which fMRI data processing and display are performed at a speed that makes them concomitant with image acquisition [1], has enabled real-time neurofeedback, i.e. allowing a person to watch and regulate the fMRI signal from his or her own brain (e.g. [2]). While earlier studies using electroencephalography (EEG) neurofeedback have demonstrated that human subjects can exert volitional control over certain EEG spectrum characteristics (e.g. [3]–[5]), rtfMRI neurofeedback has the unique advantage of precisely localizing neurophysiological activation, thus allowing focal investigations of the relationship between cognitive-behavioral function and neuroplasticity changes in deep brain structures (e.g. [6]–[8]). Recent research evidence suggests that, by using rtfMRI, individuals can learn to control neurophysiological activity in a variety of regions, including somatomotor cortex [7], [9]–[11], anterior cingulate cortex [10], [12], [13], parahippocampal cortex [6], subgenual anterior cingulate cortex [14], auditory cortex [15], and inferior frontal gyrus [16], allowing correlation between activity and function involving cognitive-behavioral domains such as somatosensory, auditory, and linguistic processing, visual perception, spatial navigation, and motor control [2], [6].

Few studies, however, have explored the feasibility of rtfMRI for training individuals to self-regulate activity in brain structures relevant to emotional processing. In one recent study, the researchers identified emotion-related networks using a functional localizer run and then asked participants (n = 13) to upregulate blood oxygen level dependent (BOLD) activity in individually (for each subject) selected region such as insula, amygdala, and ventrolateral prefrontal cortex (PFC) [17]. After a brief training period in the scanner (lasting 14 to 21 minutes), subjects were able to up-regulate the BOLD signal in these regions using negative imagery or memories and fMRI-based neurofeedback. However this study did not include a control condition in which sham feedback was provided, therefore the specificity of these results remains unclear. Other rtfMRI neurofeedback training studies demonstrated the capability to self-regulate BOLD signal in the right anterior insula [18] and the anterior cingulate cortex [13]. Finally, a previous rtfMRI neurofeedback study investigated the possibility of training healthy volunteers to control the level of BOLD activity in the amygdala during self-induced sadness, although potential learning effects were not assessed [19]. Moreover, the neurofeedback provided in this study was based on the experimenter's rating, who viewed the functional maps and then provided the individuals (n = 6) inside the MRI scanner with verbal feedback regarding the signal change in the amygdala after each trial. Although the subjects' self-ratings of mood were associated with levels of BOLD activity, the learned self-regulation could not be assessed specifically since the feedback signal and the mood induction task were always presented together. Hence, amygdala activation caused by learned self-regulation could not be differentiated from activation attributable to the mood induction.

In the present study, we investigate whether healthy volunteers could be trained to control the BOLD activity level in the amygdala by receiving rtfMRI neurofeedback while contemplating positive autobiographical memories. An extensive body of research in both humans and experimental animals has established that the amygdala plays a central role in several aspects of emotion processing, such as recognition of both positively- and negatively-valenced emotional stimuli, reward learning, and appetitive or aversive conditioning [20]–[23]. The amygdala interacts with an extended network of cortical and subcortical areas to ascribe emotional salience to events, coordinate adaptive behavioral responses to such events, and modulate the perception, attention, and memory toward emotionally-valenced stimuli [24], [25]. Recent quantitative meta-analyses of functional neuroimaging studies support a functional dissociation between left and right amygdala in terms of temporal dynamics: the right amygdala is engaged in rapid and automatic detection of emotional stimuli, while the left amygdala participates in more detailed and elaborate stimulus evaluation [20], [26], [27].

The involvement of the amygdala during mood self-induction has been reported in several studies [28]–[30]. Therefore, the possibility of volitional modulation of left amygdala activity using rtfMRI neurofeedback training provides a valuable tool to study neurophysiological regulation within neural networks involved in emotional processing. Modulation of the left amygdala with rtfMRI training might ultimately prove relevant for the development of novel therapeutic approaches for psychiatric disorders that are tractable to cognitive-behavioral interventions, such as post-traumatic stress disorder, obsessive-compulsive disorder or major depressive disorder [31]. Since the left amygdala offers a clear anatomical target that is implicated in sustained emotional processes, we implemented a mood self-induction paradigm to train healthy volunteers to control the level of hemodynamic activity in this structure. Individuals were provided with real-time fMRI neurofeedback information about their own left amygdala activity. We tested the hypothesis that healthy individuals can learn to control and voluntarily regulate the BOLD activity in their left amygdala by means of rtfMRI neurofeedback.

## Methods

### Human Subjects

Twenty-eight right-handed, medically- and psychiatrically-healthy male volunteers (age 28.0±9.0 years) participated in the study. Subjects were excluded from the study if they had: 1) current or past history of any major psychiatric disorder, 2) major medical or neurological disorders, 3) exposure to drugs likely to influence cerebral blood flow or neurological function within 3 weeks, 4) a history of drug or alcohol abuse within 1 year or a lifetime history of drug or alcohol dependence, 5) general MRI exclusion criteria. The participants' mean educational level attained was 5.3±0.9, based on the following scale: 0: no school; 1: less than 7 years of school; 2: junior high school (7th, 8th, 9th grade); 3: some high school (10th, 11th grade); 4: high school graduate (or equivalence exam); 5: some college or technical school; 6: college graduate; 7: graduate professional training (master's degree or higher). All the volunteers were naïve to fMRI neurofeedback.

The participants were randomly assigned to either an experimental group (EG, n = 14, age: 27.5±11.1 years, education: 5.1±1.0) or a control (sham) group (CG, n = 14, age: 28.4±6.6 years, education: 5.4±0.8), that were matched on age (*t*(26) = −0.27, *P*<0.790) and education (*t*(26) = −0.60, *P*<0.551). Although the control group underwent the same rtfMRI neurofeedback training as the experimental group, this group received sham rtfMRI neurofeedback information presented with rtfMRI data acquired from a different region that putatively was not involved in emotion regulation (see Regions of Interest Placement below).

The study was conducted at the Laureate Institute for Brain Research. The research protocol (protocol #: 14845) was approved by the University of Oklahoma Institutional Review Board (IRB). All the participants provided written informed consent as approved by the University of Oklahoma IRB. The subjects received financial compensation for their participation.

### Experimental Paradigm

The participants were given detailed instructions about the goal of the study and the experimental paradigm. They were instructed to retrieve positive autobiographical memories that potentially would help them learn to control the level of activity in the target brain region. Prior to scanning, each subject was asked to write down three happy autobiographical memories that could be evoked during the rtfMRI neurofeedback runs (the subjects were asked to keep details of their memories private). The participants were instructed that they would be asked to use those happy memories during scanning while attempting to increase the hemodynamic activity in the target brain region. Moreover, they would receive ongoing information about the level of neurophysiological activity in this brain area. The subjects were further instructed not to move, but instead to relax to minimize potential motion-related artifacts in the image data. Finally, it was explained to the participants that the rtfMRI neurofeedback signal is inherently delayed with respect to their mental activity by a few seconds due to the intrinsically slow hemodynamics governing the BOLD fMRI signal.

**Figure 1:**
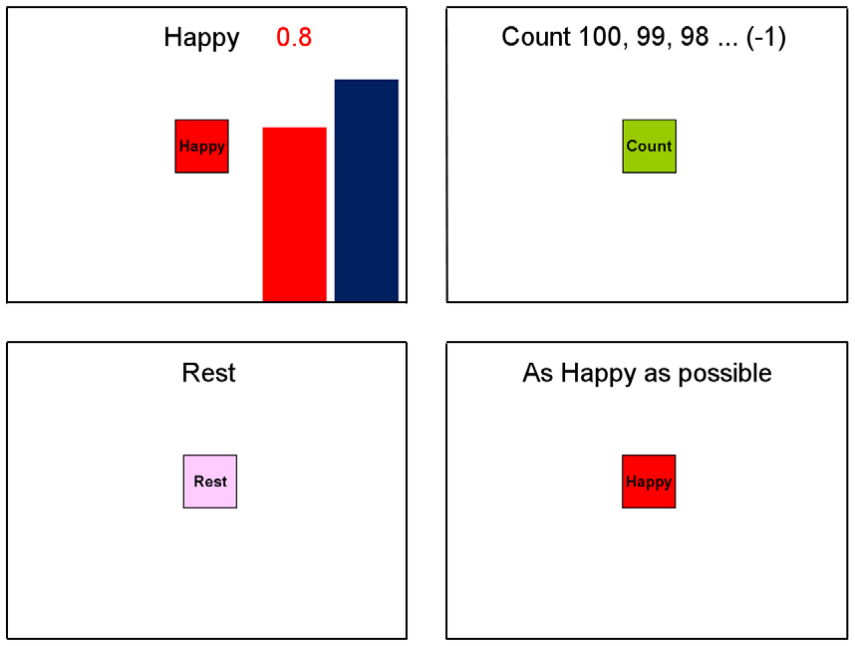
Real-time Display Screens for the Real-time fMRI Neurofeedback Procedure. Visual cues (i.e. text, color bars, and icons) were presented for each of the experimental conditions. During the Happy Memories condition, the word “Happy”, two color bars, and a number indicating the neurofeedback fMRI signal level were displayed on the screen. The participants were instructed to evoke happy autobiographical memories to make themselves feel happy while trying to increase the level of the red bar to a given target level (indicated by the fixed height blue bar). During the Count condition, the subjects saw the cue with a counting instruction, e.g. “Count 100, 99, 98 … (−1)”, and were instructed to mentally count backwards from 100 by subtracting a given integer number (shown in parentheses). During the Rest condition, the participants saw the cue “Rest” and were asked to relax while looking at the screen. For the Happy Memories condition without neurofeedback, no bars were displayed, and the cue “As Happy as possible” was presented instead.

The rtfMRI neurofeedback training paradigm included three conditions: Happy Memories, Count, and Rest (Fig. 1). For each condition, cues were presented on the screen using both text and color icons to indicate each condition. During the *Happy Memories condition* involving neurofeedback, the cue “Happy” and two color bars (red, blue) were displayed on the screen. The red bar represented the actual neurofeedback signal, which was updated continuously by changing the height of the bar either upward or downward based on the corresponding level of BOLD activity. This neurofeedback signal was also indicated by a number shown above the red bar. The participants were instructed to retrieve and contemplate the positive autobiographical memories while also attempting to increase the level of the red bar to that of the fixed target level displayed by the blue bar. Because the Happy Memories condition required memory recall and rumination on those memories could potentially not be stopped quickly [19], [32], and as our preliminary experiments had indicated that a single control condition was insufficient, two control conditions were implemented to distract the subjects' attention from contemplating positive memories and to dampen the activation of the emotion regulation network [33]. During the *Count condition*, the subjects were shown the cue with a specific instruction to count backwards from 100 by a subtracting a specified integer. This number was 1, 2, 3, and 4 for Run 1, Run 2, Run 3, and the Transfer run, respectively (see Experimental Protocol below). During the subsequent *Rest condition*, the participants were presented with the cue “Rest” and were asked to relax and breathe regularly while looking at the display screen (Fig. 1). No bars were displayed during the Count and Rest conditions. Similarly, no bars were shown for the Happy Memories condition without neurofeedback (during the Transfer run, see below), during which the instruction cue read “As Happy as possible”.

A subset of the participants (n = 6) agreed after the session to provide a general account (without revealing any specific details) of the memories that were most effective at helping them to feel happy and raise the neurofeedback bar level. Based on their reports, two categories of positive autobiographical memories appeared to work best for the purpose of the present study: memories involving close family members and memories of specific joyful events (e.g., vacations, weddings, other celebrations, and similar).

### Experimental Protocol

**Figure 2:**
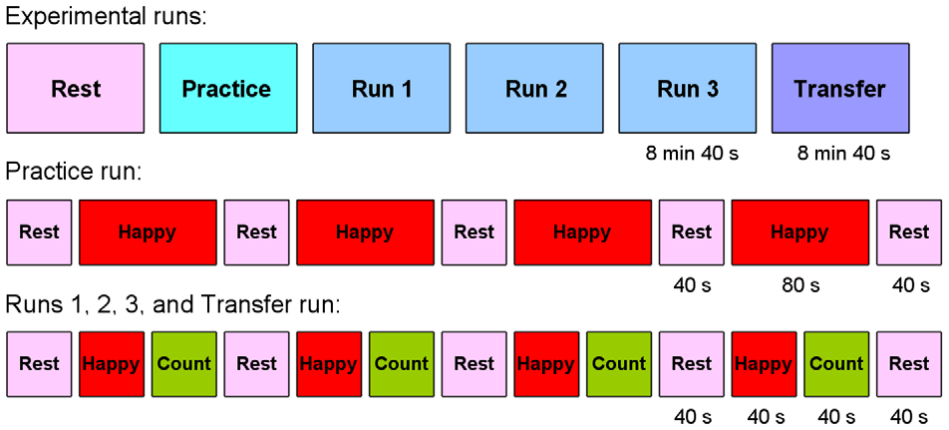
Protocol for the Real-time fMRI Neurofeedback Experiment. The experimental procedure consisted of six runs each lasting 8 min 40 sec. During the Rest run, the participants were instructed to rest. During the Practice run, the subjects were given the opportunity to become comfortable with the rtfMRI neurofeedback procedure. During Runs 1, 2, and 3, the participants underwent rtfMRI neurofeedback training consisting of alternating blocks of Rest, Happy, and Count conditions, each lasting 40 seconds. During the Transfer Run, the subjects were instructed to perform the same task as during the neurofeedback training, but neurofeedback information (bars, number) was not be provided.

The rtfMRI neurofeedback experiment consisted of six fMRI runs each lasting 8 minutes 40 seconds (Fig. 2). During the first *Rest* run (RE), a resting state paradigm was employed, and the participants were instructed to let their minds wander while fixating at the display screen. During the second *Practice* run (PR), the subjects were given an opportunity to become comfortable with the rtfMRI neurofeedback procedure. The Practice run consisted of alternating blocks of Rest (5 blocks lasting 40 seconds each) and Happy Memories (4 blocks lasting 80 seconds each) conditions (Fig. 2). For the first three Happy Memories condition blocks, the participants were instructed to recall and contemplate the prepared positive autobiographical memories, and then, for the last Happy Memories condition block, to use the one memory that elevated their mood to the greatest extent. Thus, the Practice run allowed the subjects (i) to accommodate to the neurofeedback condition; (ii) to evaluate the emotional impact of the three prepared happy memories within the experimental setting; and (iii) to practice switching from one memory to another during the neurofeedback training. During the subsequent three fMRI runs — *Run 1* (R1), *Run 2* (R2), and *Run 3* (R3) — the participants underwent the rtfMRI neurofeedback training as they were instructed during the pre-training session. Those three runs consisted of alternating blocks of Rest (5 blocks), Happy Memories (4 blocks), and Count (4 blocks) conditions, each lasting 40 seconds (Fig. 2). The subjects were encouraged to try various other happy autobiographical memories if the currently-chosen one did not help them raise the red bar during the neurofeedback training. The participants were presented with a target activation level (blue bar), which they were asked to attempt to match during the Happy Memories condition blocks. Because our preliminary experiments had indicated that the activation level of the left amygdala could be as high as 2% BOLD signal change in some subjects, the target level was set to 0.5%, 1.0%, 1.5%, and 2.0% for the Practice run, Run 1, Run 2, and Run 3, respectively. Finally, during the *Transfer run* (TR), the participants were instructed to perform the same task as during the neurofeedback training, but rtfMRI neurofeedback information was not provided for the blocks of the Happy Memories condition and the bars were not shown. The Transfer run was performed to assess the transfer of the learned control and to check whether the training effect generalized to situations where no feedback was available.

Before the rtfMRI neurofeedback training, the subjects were asked to complete the 20-item Toronto Alexithymia Scale (TAS-20), as well as the 15-item Emotional Contagion (EC) Scale. TAS-20 assesses an individual's difficulty in understanding, processing, or describing emotions [34], [35]. This self-report instrument consists of 3 subscales: (i) Difficulty Identifying Feelings subscale (7 items), (ii) Difficulty Describing Feelings subscale (5 items), and the Externally Oriented Thinking subscale (8 items). The EC scale assesses an individual's susceptibility to other people's emotions [36]. It includes five subscales for five basic emotions: Love (items 6, 9, 12), Happiness (items 2, 3, 11), Fear (items 8, 13, 15), Anger (items 5, 7, 10), and Sadness (items 1, 4, 14).

### Regions of Interest Placement

**Figure 3:**
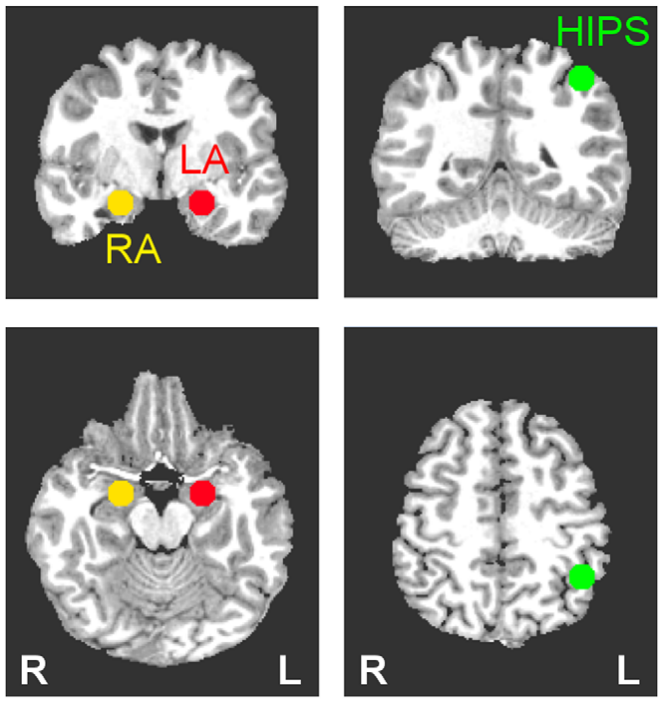
Regions of Interest (ROIs) for the Real-time fMRI Neurofeedback Procedure. Three regions of interest (spheres of 7 mm radius) were used to assess changes in BOLD activity in the left amygdala (LA, red), right amygdala (RA, yellow), and left horizontal segment of the intraparietal sulcus (HIPS, green). The ROI placements are illustrated on T1-weighted coronal (upper row) and axial (lower row) human brain sections in the Talairach space. Following the radiological notation, the left side (L) of the brain is shown on the right, and the right side (R) of the brain – on the left.

The rtfMRI neurofeedback procedure was based on an MRI-based region-of-interest (ROI) approach. Three ROIs were defined as spheres of 7 mm radius in the stereotaxic array of Talairach and Tournoux [37] and placed, respectively, in the *left amygdala* (LA: −21, −5, −16), *right amygdala* (RA: 21, −5, −16), and *left horizontal segment of the intraparietal sulcus* (HIPS: −42, −48, 48), as illustrated in Figure 3. The specified ROI centers were chosen based on quantitative meta-analyses of functional neuroimaging studies investigating either the role of LA and RA in emotional processing [20] or the role of HIPS in number processing [38]. The neurofeedback signal was based on fMRI activation in the left amygdala ROI for the participants in the experimental group and on the fMRI activation in the left HIPS ROI for the subjects in the control (sham) group.

### Data Acquisition

All functional and structural MR images were collected at the Laureate Institute for Brain Research using a General Electric Discovery MR750 whole-body 3 Tesla MRI scanner. The scanner is equipped with a scalable 32-channel digital MRI receiver capable of performing massively-parallel fMRI in real time [39]. A standard 8-channel receive-only head coil array was used for MRI signal reception. The MR750 scanner is also equipped with a custom developed real-time MRI/fMRI system [40], which made it possible to implement rtfMRI neurofeedback. With this system, imaging hardware, and ultra-fast Echo Planar Imaging (EPI) sequence used, real-time fMRI acquisition is easily maintained. The instrumentation delays due to image reconstruction, image transfer, real-time data processing, network communications, computer image processing, and neurofeedback GUI display totaled less than one second.

A single-shot gradient-recalled EPI sequence with Sensitivity Encoding (SENSE) [41] was employed for fMRI. The following EPI imaging parameters were used: FOV/slice = 240/2.9 mm, axial slices per volume = 34, acquisition matrix = 96×96, repetition/echo time *TR*/*TE* = 2000/30 ms, SENSE acceleration factor *R* = 2 in the phase encoding (anterior-posterior) direction, flip angle = 90°, sampling bandwidth = 250 kHz, number of volumes = 263. Each functional scan time lasted 8 min 40 sec. Three EPI volumes (6 sec) were added at the beginning of each fMRI run to allow the fMRI signal to reach steady state, and were excluded from data analysis. The EPI images were reconstructed into a 128×128 matrix, in which the resulting fMRI voxel volume was 1.875×1.875×2.9 mm^3^. Additionally, simultaneous physiological pulse oximetry [42] and respiration waveform recordings were conducted (with 50 Hz sampling) for each fMRI run. A photoplethysmograph with an infra-red emitter placed under the pad of the subject's left index finger was used for pulse oximetry, and a pneumatic respiration belt was used for respiration measurements. A T1-weighted magnetization-prepared rapid gradient-echo (MPRAGE) sequence with SENSE was used to provide an anatomical reference for the fMRI analysis. It had the following parameters: FOV = 240 mm, axial slices per slab = 128, slice thickness = 1.2 mm, image matrix = 256×256, *TR*/*TE* = 5/1.9 ms, acceleration factor *R* = 2, flip angle = 10°, delay time *TD* = 1400 ms, inversion time *TI* = 725 ms, sampling bandwidth = 31.2 kHz, scan time = 4 min 58 sec.

### Data Processing and Analysis

The image data analyses were performed using Analysis of Functional NeuroImages (AFNI, http://afni.nimh.nih.gov/) [43] software within the framework of the General Linear Model (GLM) [44]. Statistical data analyses were carried out using Statistical Package for Social Sciences (IBM SPSS Statistics 19, http://www-01.ibm.com/software/analytics/spss) and MATLAB Statistics Toolbox (MathWorks Inc, http://www.mathworks.com/).

The neurofeedback was implemented using the custom real-time fMRI system [40] utilizing the real-time features of AFNI [45] and a custom developed graphic user interface (GUI) software. The three ROIs, defined as described above, were transformed to the EPI image space using each subject's high-resolution MPRAGE structural data. The resulting ROIs in the EPI space contained approximately 140 voxels each. In our neurofeedback implementation, the AFNI real-time plug-in was used to perform volume registration of EPI images and to export mean values of fMRI signals for the three ROIs in real time. The first three volumes of each experimental run were excluded to allow the fMRI signal to reach steady state. The rtfMRI signal for each Happy Memories condition was measured as a percent signal change relative to the baseline obtained by averaging the fMRI signal for the preceding 40-sec long Rest condition block. This neurofeedback signal (percent signal change) was updated every 2 sec and displayed on the screen as the red bar. To reduce bar fluctuations due to noise in the fMRI signal, the bar height was computed at every time point as a moving average of the current and two preceding fMRI percent signal change values. Our preliminary experiments had indicated that the neurofeedback bar fluctuations caused by fMRI noise could be a distraction factor, preventing the subject from focusing on the emotion self-induction task. Implementation of the moving average for the neurofeedback signal reduced this problem. While this approach reduced the effective temporal resolution of the neurofeedback procedure to some extent, it did not pose any limitations for the present study because the positive autobiographical memory retrieval and related changes in BOLD fMRI signal in the left amygdala region were associated with considerably longer time scales.

To determine whether observed training effects reflected the participants' learning to volitionally control brain activation using rtfMRI neurofeedback, the subjects in the control (sham) group, unaware that they were presented with the sham neurofeedback, performed an identical training sequence. The sham information consisted of fMRI data derived from the left HIPS ROI instead of the left amygdala ROI and, therefore, was not expected to correlate with the performance in the mood self-induction task.

Pre-processing of single-subject fMRI data included correction of cardiorespiratory artifacts using AFNI implementation of the RETROICOR method [46]. The cardiac and respiratory waveforms recorded simultaneously during each fMRI run were used to generate the cardiac and respiratory phase time series for the RETROICOR. Further fMRI pre-processing included volume registration and slice timing correction for all EPI volumes in a given exam. Standard GLM analysis was then applied separately for each of the six fMRI runs. The following regressors were included in the GLM model: two block stimulus conditions (Happy Memories, Count), six motion parameters as nuisance covariates to take into account possible artifacts caused by head motion, and five polynomial terms for modeling the baseline. The stimulus conditions for all runs (including the Rest and Practice runs) consisted of 40-second-long blocks as defined for Runs 1–3 and the Transfer run in Figure 2. Hemodynamic response amplitudes were estimated using the standard regressors, constructed by convolving a boxcar function (representing the block duration) with the canonical hemodynamic response function using standard AFNI parameters. The GLM *ß* coefficients were computed for each voxel using the 3dDeconvolve AFNI program and then converted to percent signal changes for Happy versus Rest, Count versus Rest, and Happy versus Count contrasts. The resulting fMRI percent signal change maps for each run were spatially transformed to the stereotaxic array of Talairach and Tournoux [37] and re-sampled to 2×2×2 mm^3^ isotropic voxel size. They were subsequently used for whole-brain statistical group analyses. The voxel-wise percent signal change data were also averaged within the three ROIs (LA, RA, HIPS) and used as a performance measure.

In preparation for the whole-brain statistical group analysis, the spatially-normalized fMRI percent signal change maps were spatially smoothed using a Gaussian kernel with full width at half maximum (FWHM) of 5 mm. Group *t*-tests comparing the percent signal change data to zero activation level were employed to generate statistical activation maps for the Happy versus Rest, Count versus Rest, and Happy versus Count contrasts. The statistical activation maps were corrected for multiple comparisons using the false discovery rate (FDR) approach [47]. Group *t*-test was also applied to Happy versus Rest activation data for the experimental and control (sham) groups to examine statistical differences between the groups.

Inferential statistical analyses were applied to the average activation results for the three ROIs. First, the training effect was evaluated by applying a three-way 4 (Training)×2 (ROI)×2 (Group) ANOVA for repeated measures on percent signal changes with Training (PR, R1, R2, R3) and ROI (LA, HIPS) as within-subjects factors and Group (EG, CG) as a between-subjects factor. Second, specificity of the training effect to the LA ROI was evaluated (within each group) by applying a two-way 4 (Training)×2 (ROI) ANOVA for repeated measures on percent signal changes with Training (PR, R1, R2, R3) and ROI (LA, HIPS) as within-subjects factors. Third, specificity of the training effect to the experimental group was evaluated (for each ROI) by using a two-way 4 (Training)×2 (Group) ANOVA for repeated measures with Training (PR, R1, R2, R3) as a within-subject factor and Group (EG, CG) as a between-subjects factor. Fourth, monotonic properties of the participants' control over brain activation across all runs were evaluated (for each ROI and Group) by using a one-way ANOVA trend analysis for repeated measures on percent signal changes with Time (RE, PR, R1, R2, R3, TR) as a within-subjects factor. Finally, generalization of the training effect beyond the actual training was evaluated (for the LA and RA ROIs) by applying a paired *t*-test for percent signal changes between the Transfer run (TR) and the last training run (R3). Associations between average percent signal changes for LA ROI and sub-scores of the TAS-20 and EC psychological scales were determined using Pearson bivariate correlations.

To determine functional connectivity of the amygdala network, a GLM-based functional connectivity analysis was applied using a seed ROI in the left amygdala region. The seed ROI was defined as a sphere of 5 mm radius in the Talairach space. After transformation to an individual subject's EPI image space, this ROI contained approximately 50 voxels. The volume-registered and slice-timing-corrected single-subject fMRI data from each run were low-pass filtered at 0.08 Hz. The time course of the mean fMRI signal from the seed ROI was used as a stimulus regressor. The GLM model for each run also included six motion parameters, five polynomial terms for modeling the baseline, and time courses from two additional ROIs defined, respectively, within the deep white matter and the CSF of the lateral ventricles. The GLM-based *R*-squared statistics were converted to correlation coefficient values *r* and the resulting correlation maps for each run were transformed to the Talairach space, re-sampled, and spatially smoothed (5 mm FWHM). For statistical analyses, the correlation coefficient values were converted to *z* scores using the Fisher *r*-to-*z* transformation. Group *t*-test with respect to zero level was employed to determine the functional connectivity pattern for each run. Correction for multiple comparisons was based on FDR.

To identify regions within the network, for which the functional connectivity with the left amygdala increased during the experiment, mean values of the correlation coefficients for several spherical ROIs (of 5 mm radius) were determined for each run. The ROIs were centered at locations that were characterized by peak *t* values in the statistical group connectivity analysis of the experimental group for the Transfer run. For each of these ROIs, a one-way ANOVA trend analysis for repeated measures on mean correlation coefficients was applied (for each group) with Training (PR, R1, R2, R3, TR) as a within-subjects factor.

## Results

### ROI Analysis

**Figure 4:**
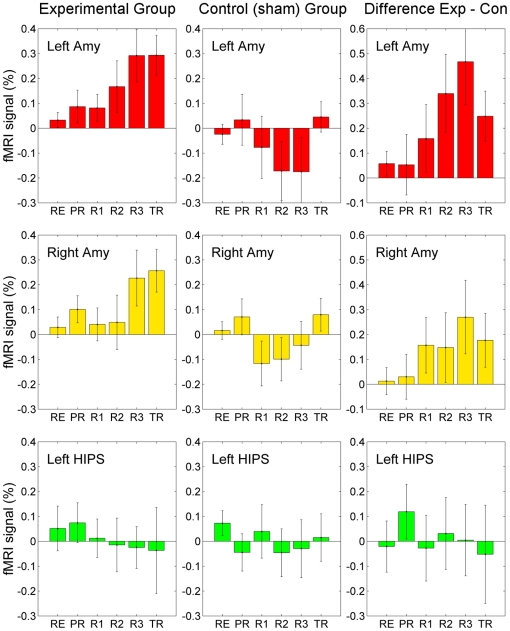
Learned Enhancement of Control over BOLD fMRI Activation and Mood Induction. A significant training effect was observed for the left amygdala for the subjects in the experimental group. The control of BOLD fMRI activation in the left amygdala ROI monotonically increased over training runs and persisted during the Transfer run. Each bar represents mean percent signal change in the BOLD signal (± s.e.m.) averaged across Happy Memories conditions during a given run (see text for details) for each ROI (left amygdala, red; right amygdala, yellow; left HIPS, green) and group (experimental, control). The difference between the corresponding average fMRI percent signal change values for the experimental and control (sham) groups is also shown.

Results of the neurofeedback experiment based on the ROI analysis are exhibited in Figure 4. Each bar in the figure represents a mean fMRI percent signal change for a given ROI, averaged for Happy Memories conditions during a given run and across all subjects in a given group. The mean ROI results for each participant were obtained from the GLM analysis using the same stimulus regressors for each run (including the Rest and Practice runs, see Data Processing and Analysis for details). The error bars are standard errors of the means (s.e.m.). The data show that the average BOLD activity in the LA ROI increased progressively across the neurofeedback runs for the experimental group and reached a maximum during the final neurofeedback run (Run 3). The subsequent Transfer run was characterized by a similar activation level (Fig. 4, left). For the control (sham) group, the average fMRI activation level for the left amygdala ROI decreased across the neurofeedback runs and reached its minimum during Run 3 (Fig. 4, middle). The difference between the average activation levels for the two groups exhibited a steady increase across the neurofeedback runs and exceeded 0.4% for Run 3 (Fig. 4, right). These results demonstrate the ability of the participants in the experimental group to regulate BOLD activity of their left amygdala using rtfMRI neurofeedback. Notably these results are based on average values for all Happy Memories conditions within a given run, while activation levels at a given Happy moment could be considerably higher. The results for the right amygdala ROI in Figure 4 also demonstrate an increase in the average BOLD activity for the training runs in the experimental group. The increase, however, is less pronounced than for the left amygdala ROI. The BOLD activation levels for the left HIPS ROI are close to zero (after group averaging) and exhibit no obvious trend across runs (Fig. 4).

Several statistical tests were performed to evaluate the data in Figure 4 (see Data Processing and Analysis) and provided the following results. First, the three-way 4 (Training: PR, R1, R2, R3)×2 (ROI: LA, HIPS)×2 (Group: EG, CG) ANOVA revealed non-significant main effects for Training (*F*(3,78) = 0.52, *P*<0.669), ROI (*F*(1,26) = 0.16, *P*<0.689), and Group (*F*(1,26) = 3.38, *P*<0.078), and a non-significant interaction effects for Training×Group (*F*(3,78) = 0.73, *P*<0.540), Training×ROI (*F*(3,78) = 0.51, *P*<0.679), and ROI×Group (*F*(1,26) = 2.33, *P*<0.139). However, a *significant* three-way interaction effect was evident for Training×ROI×Group (*F*(3,78) = 3.73, *P*<0.015). This result suggests that the experimental and control (sham) groups differed in their neurofeedback training effects based on the specific target brain region (LA, HIPS).

Second, the two-way 4 (Training: PR, R1, R2, R3)×2 (ROI: LA, HIPS) ANOVA for the experimental group (EG) showed a non-significant main effect for Training (*F*(3,39) = 0.38, *P*<0.766), but a *significant* effect for ROI (*F*(1,13) = 10.28, *P*<0.007), and a *significant* interaction effect for Training×ROI (*F*(3,39) = 3.05, *P*<0.040). For the control group (CG), in contrast, non-significant main and interaction effects (Training: *F*(3,39) = 0.95, *P*<0.426; ROI: *F*(1,13) = 0.35, *P*<0.567; Training×ROI: *F*(3,39) = 1.31, *P*<0.287) were found. These results indicate a significant training effect in the target region (LA) for the experimental group.

Third, the two-way 4 (Training: PR, R1, R2, R3)×2 (Group: EG, CG) ANOVA for the LA ROI revealed a non-significant main effect for Training (*F*(3,78) = 0.55, *P*<0.652), but a *significant* main effect for Group (*F*(1,26) = 4.71, *P*<0.039) as well as a *significant* interaction effect for Training×Group (*F*(3,78) = 3.05, *P*<0.033). Independent *t*-tests of the LA ROI activations for EG and CG for each of the six runs (RE: *t*(26) = 1.16, *P*<0.257; PR: *t*(26) = 0.60, *P*<0.557; R1: *t*(26) = 1.17, *P*<0.254; R2: *t*(26) = 2.16, *P*<0.040; R3: *t*(26) = 2.70, *P*<0.012; TR: *t*(26) = 2.47, *P*<0.020) showed *significant* differences in mean LA ROI activation levels between the two groups for Run 2, Run 3, and the Transfer run. A similar two-way 4×2 ANOVA analysis for the RA ROI revealed a non-significant main effect for Training (*F*(3,78) = 2.11, *P*<0.105), a marginally significant main effect for Group (*F*(1,26) = 3.81, *P*<0.062), and a non-significant interaction effect for Training×Group (*F*(3,78) = 0.44, *P*<0.722). A marginally significant difference in mean RA ROI activations between EG and CG was found for Run 3 (RE: *t*(26) = 0.24, *P*<0.814; PR: *t*(26) = 0.88, *P*<0.388; R1: *t*(26) = 1.41, *P*<0.171; R2: *t*(26) = 1.06, *P*<0.300; R3: *t*(26) = 1.83, *P*<0.079; TR: *t*(26) = 1.63, *P*<0.115). These results demonstrate that the experimental and control groups differed significantly in the neurofeedback training effects on LA activation, and less significantly – in the effects on RA activation.

Fourth, one-way ANOVA trend analysis across all runs (RE, PR, R1, R2, R3, TR) for the LA ROI activation showed a *significant* linear trend (*F*(5,65) = 2.23, *P*<0.062; Linear: *F*(1,13) = 11.00, *P*<0.006; Quadratic: *F*(1,13) = 0.09, *P*<0.767; Cubic: *F*(1,13) = 0.12, *P*<0.734) for the experimental group, but not for the control group (*F*(5,65) = 1.51, *P*<0.198; Linear: *F*(1,13) = 0.30, *P*<0.595; Quadratic: *F*(1,13) = 1.26, *P*<0.281; Cubic: *F*(1,13) = 5.48, *P*<0.036). A marginally significant linear trend across all runs was found for the RA ROI activation in the experimental group (*F*(5,65) = 1.78, *P*<0.130; Linear: *F*(1,13) = 5.00, *P*<0.043; Quadratic: *F*(1,13) = 0.85, *P*<0.374; Cubic: *F*(1,13) = 0.63, *P*<0.442). In contrast, no significant trend was found for the HIPS ROI in the experimental group (*F*(5,65) = 0.34, *P*<0.886; Linear: *F*(1,13) = 0.54, *P*<0.474; Quadratic: *F*(1,13) = 0.001, *P*<0.980; Cubic: *F*(1,13) = 0.50, *P*<0.494). These results indicate a monotonic increase in activations of both LA and RA across all runs for the experimental group.

Finally, the paired *t*-test of the LA ROI activation levels for the the Transfer run vs. the last neurofeedback training run (TR vs. R3) within the experimental group showed no difference in activation means (*t*(13) = 0.01, *P*<0.992). A similar effect was found for the RA ROI activation (*t*(13) = 0.31, *P*<0.765). These results suggest that the neurofeedback training effects in both LA and RA persisted during the Transfer run (without neurofeedback).

**Figure 5:**
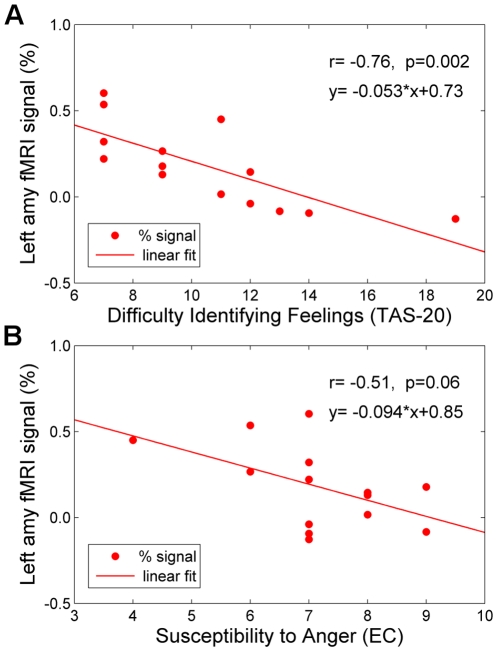
Relationship between the Neurofeedback Training Effect on the Left Amygdala Activation and Individual Psychological Scores. **A) Correlation with the Difficulty Identifying Feelings (TAS-20).** The training effect for the left amygdala was correlated with the participants' insight into their feelings. Thus the more highly the participants rated their capacity for identifying their own feelings (based on the Difficulty Identifying Feelings sub-scale of the Toronto Alexithymia Scale, TAS-20), the more they increased the BOLD signal in the left amygdala during training. **B) Correlation with the Susceptibility to Anger (EC).** The higher the participants rated their sensitivity to other peoples' anger (based on the Susceptibility to Anger sub-scale of the Emotional Contagion scale, EC), the less BOLD activation was observed in their left amygdala during training. The activation levels shown (in both A and B) are averages across the three neurofeedback training runs (Runs 1–3) for each subject in the Experimental group.

Figure 5 illustrates an association between the neurofeedback performance of the subjects in the experimental group and their ability to identify feelings (measured by the TAS-20 scale), as well as their susceptibility to anger (measured by the EC scale). Each data point in the figure is the subject's percent change in BOLD signal in the LA, averaged across three neurofeedback training runs (R1, R2, R3). Pearson bivariate correlations were applied to investigate the relations between the average LA activity and the sub-scores of TAS-20 and EC. A *significant* negative correlation with the Difficulty Identifying Feelings score (DIF: *r* = −0.76, *P*<0.002) of TAS-20 was found (Fig. 5A), indicating that a greater difficulty in identifying feelings was associated with a diminished ability to self-regulate LA BOLD fMRI activity during the neurofeedback training. No significant correlation was observed with the Difficulty Describing Feelings score (DDF: *r* = 0.15, *P*<0.615) or the Externally Oriented Thinking score (EOT: *r* = −0.25, *P*<0.396). Note that the experimental and control groups did not differ in any of these psychological measures (DIF: *t*(26) = 0.71, *P*<0.482; DDF: *t*(26) = 1.34, *P*<0.193; EOT: *t*(26) = 0.12, *P*<0.908). Negative correlation was also found between the left amygdala activation and the Susceptibility to Anger score (*r* = −0.51, *P*<0.061) for the experimental group (Fig. 5B), though the result is only marginally significant. Note also that the Difficulty Identifying Feelings score (TAS-20) and the Susceptibility to Anger score (EC) were themselves uncorrelated (*r* = 0.10, *P*<0.722, for the 14 subjects in the experimental group).

**Figure 6:**
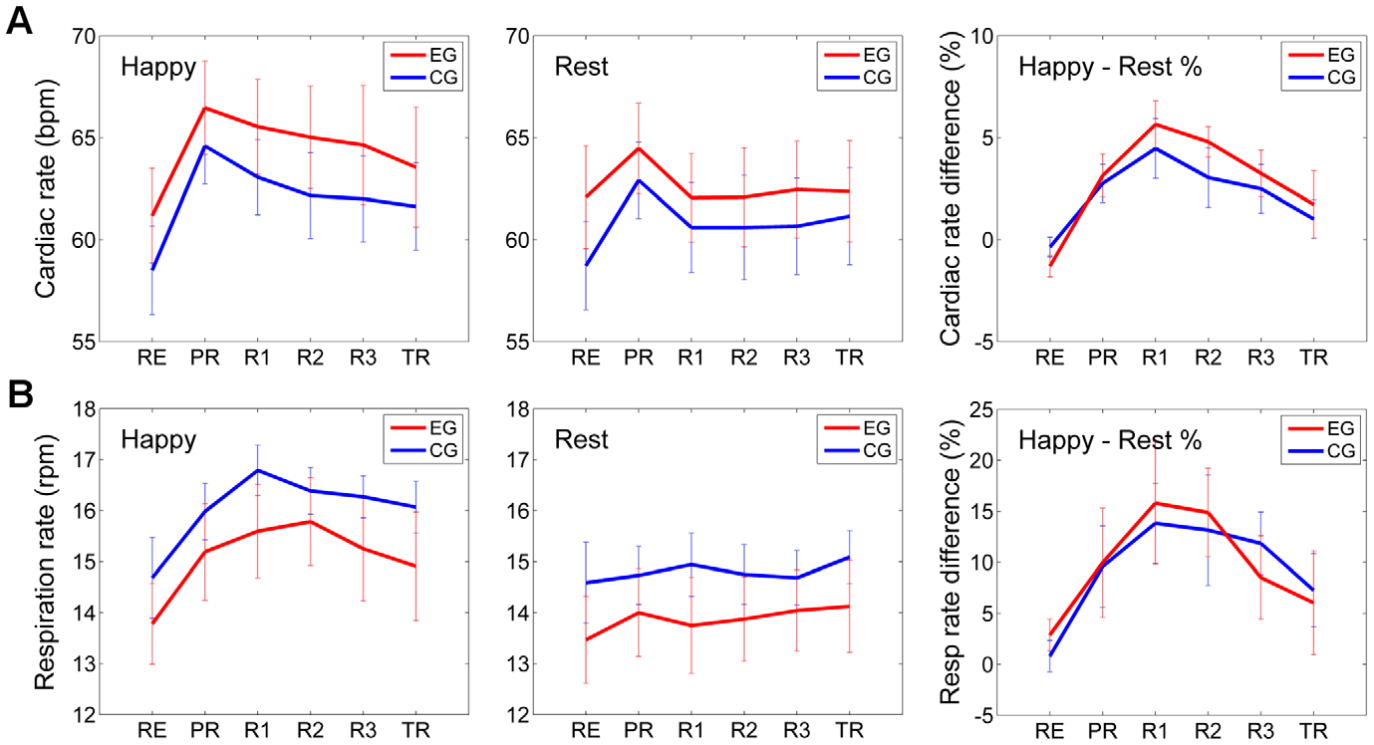
Cardiac and Respiratory Rate Variations during the Neurofeedback Experiment. **A) Average Cardiac Rate.** The experimental group (EG, red) and control (sham) group (CG, blue) exhibited no statistically significant differences in mean cardiac rates for either Happy Memories or Rest conditions for any of the six experimental runs. **B) Average Respiratory Rate**. Similarly, no statistically significant differences were observed in mean respiratory rates of the two groups (EG and CG) for either condition for any of the runs.

Figure 6 describes variations in cardiac (A) and respiratory (B) rates during the neurofeedback experiment for the participants in the experimental (red) and control (blue) groups. The results in Figure 6A are based on cardiac data for 13 subjects from EG and 12 subjects from CG (cardiac recording from the other subjects in the two groups were unusable due to technical issues). Each data point for the Happy Memories condition represents cardiac rate averaged across four 40-second-long Happy Memories condition blocks (Fig. 2) in a given run and for all the subjects in a given group. Each bar for the Rest condition is an average cardiac rate across four 40-sec long Rest blocks, preceding the Happy Memories blocks in a given run for all the subjects in a given group. For the Rest and Practice runs, the “Happy Memories” and “Rest” condition blocks were defined in the same way as for Runs 1–3 and the Transfer run in Figure 2 (see Data Processing and Analysis). According to Figure 6A, the average cardiac rate for the Happy Memories condition increased significantly for both experimental and control groups during the Practice run, when the participants were first exposed to rtfMRI neurofeedback, and then decreased gradually as training progressed. However, no significant difference in mean cardiac rates between the two groups was observed for the Happy Memories condition for any of the six experimental runs (RE: *t*(1,23) = 0.84, *P*<0.409; PR: *t*(1,23) = 0.63, *P*<0.538; R1: *t*(1,23) = 0.82, *P*<0.418; R2: *t*(1,23) = 0.87, *P*<0.396; R3: *t*(1,23) = 0.72, *P*<0.476; *t*(1,23) = 0.52, *P*<0.607).

The respiration rate results in Figure 6B were analyzed and presented in the same way as the cardiac rate results in Figure 6A. The two groups in this case included, respectively, 12 subjects from EG and 13 subjects from CG. Figure 6B demonstrates that the average respiration rate for the Happy Memories condition has a broad maximum, and peaks during Run 1 for the control group and during Run 2 for the experimental group. However, as with the cardiac rate results, no significant difference in mean respiration rates between the two groups was found for the Happy Memories condition for any of the experimental runs (RE: *t*(1,23) = −0.81, *P*<0.428; PR: *t*(1,23) = −0.74, *P*<0.468; R1: *t*(1,23) = −1.17, *P*<0.252; R2: *t*(1,23) = −0.63, *P*<0.534; R3: *t*(1,23) = −0.95, *P*<0.351; *t*(1,23) = −1.01, *P*<0.323). Note that both cardiac and respiratory rates for the Happy Memories condition exhibit the largest relative (%) increase over the corresponding rates for the Rest condition during Run 1 in both groups (Fig. 6, right).

### Whole-brain Activation Analysis

**Figure 7:**
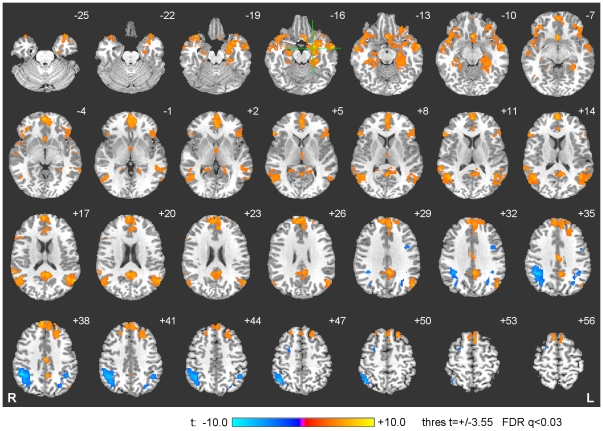
Activation Network for Happy Memories and Count Conditions. The group activation analysis for Happy>Count contrast revealed significant BOLD signal changes in a fronto-temporo-limbic network, while the Count>Happy contrast revealed activations in a parietal network (see text for details and Table 1 for coordinates). The activation maps are projected on a representative single-subject T1 template in the Talairach space with 3 mm separation between axial slices (the number adjacent to each slice indicates the *z* coordinate in mm from the bicommissural plane, with positive z indicating dorsal). The left hemisphere (L) is to the reader's right. The green crosshairs mark the center of the left amygdala ROI.

Results of group activation analysis of the fMRI data for the experimental group are shown in Figure 7, which exhibits a statistical activation map for Happy versus Count contrast for the Transfer run. Parameters of the activation centers in Figure 7 are specified in Table 1. The activation centers were identified using the cluster technique with the significance threshold set to FDR *q*<0.03 and the minimum cluster size set to 20 voxels. The Happy>Count contrast reveals activations in a fronto-temporo-limbic network including bilateral superior frontal gyrus (SFG), bilateral ventrolateral prefrontal cortex (VLPFC), right medial frontal polar cortex (MFPC), right dorsomedial prefrontal cortex (DMPFC), and right ventromedial prefrontal cortex (VMPFC) in the frontal lobe; bilateral superior temporal gyrus (STG) and bilateral middle temporal gyrus (MTG) in the temporal lobe; and left amygdala (encompassing the LA ROI), bilateral hippocampus (HC), left parahippocampal gyrus (PHG), left pregenual anterior cingulate cortex (ACC), left posterior cingulate cortex (PCC), and right subgenual anterior cingulate cortex (ACC) in the limbic lobe. The Count>Happy contrast revealed activations in bilateral inferior parietal lobule (IPL, encompassing the left HIPS ROI) and left parieto-occipital transition cortex in the parietal lobe.

**Table 1:** Activation Network for Happy Memories and Count Conditions.

		Talairach coordinates			
Region	Laterality	x	y	z	t-score*
**Frontal Lobe**	**Happy Memories>Count**				
Ventrolateral prefrontal cortex (BA 45)	L	−53	25	10	5.5
Superior frontal gyrus (BA 8)	L	−21	27	52	6.4
Ventrolateral prefrontal cortex (BA 45)	R	53	21	12	6.2
Superior frontal gyrus (BA 6)	R	7	21	56	6.1
Medial frontal polar cortex (BA 9/10)	R	4	56	26	7.2
Dorsomedial prefrontal cortex (BA 8)	R	7	47	40	8.6
Medial frontal polar cortex (BA 10)	R	2	55	16	7.4
Ventromedial prefrontal cortex (BA 10)	R	2	51	−5	8.1
**Temporal Lobe**	**Happy Memories>Count**				
Superior temporal gyrus (BA 39)	L	−57	−61	20	8.2
Middle temporal gyrus (BA 21)	L	−67	−45	−2	6.1
Middle temporal gyrus (BA 21)	L	−59	−5	−16	9.4
Superior temporal gyrus (BA 22)	R	57	−43	16	7.5
Superior temporal gyrus (BA 22)	R	45	7	−10	6.2
Middle temporal gyrus (BA 21/37)	R	44	−58	12	7.6
Middle temporal gyrus (BA 21)	R	57	−7	−12	5.7
**Limbic Lobe**	**Happy Memories>Count**				
Amygdala	L	−23	−3	−16	8.7
Parahippocampal gyrus (BA 36)	L	−28	−29	−17	7.1
Hippocampus	L	−22	−14	−14	7.1
Pregenual anterior cingulate cortex (BA 24)	L	−1	39	3	6.6
Posterior cingulate cortex (BA 30)	L	−5	−51	18	7.2
Hippocampus	R	26	−13	−17	5.4
Subgenual anterior cingulate cortex (BA 25)	R	1	17	−8	9.6
**Parietal Lobe**	**Count>Happy Memories**				
Inferior parietal lobule (BA 40)	L	−43	−51	42	−6.0
Parieto-occipital transition cortex	L	−27	−71	42	−4.7
Inferior parietal lobule (BA 40)	R	43	−47	38	−9.9

Changes in regional BOLD activity associated with the Happy Memories and Count conditions for the Transfer run.

BA – Brodmann areas; L – left; R – right.

*q(FDR)<0.03 (minimum 20 voxels).

To compare activation results between the experimental and control groups, a group *t*-test was applied to the Happy versus Rest percent signal change data from the two groups. The test results for the Transfer run are summarized in Table 2. Because FDR correction for multiple comparisons over the whole brain yielded results below the statistical significance level in this case, the uncorrected results thresholded at *p*<0.05 (*t* = ±2.057) are reported, with the minimum cluster size set to 20 voxels. According to Table 2, a number of brain regions exhibit higher BOLD activations during the Happy Memories task for the experimental group than for the control group. They include: left SFG, right VLPFC, and right VMPFC in the frontal lobe; bilateral MTG in the temporal lobe; bilateral amygdala, bilateral PHG, left pregenual ACC, right periamygdaloid cortex, and right posterior cingulate cortex in the limbic lobe. Two regions – left VLPFC (BA 44) and right middle frontal gyrus (BA 8) – show higher activations for the control group than for the experimental group. The largest difference between the two groups in the left amygdala region occurs at the locus (−17, −7, −16), close to the boundary of the left amygdala and the hippocampus, which both play a role in emotional memory retrieval [48].

**Table 2:** Comparison of Activations between Experimental and Control Groups for Happy Memories Condition.

		Talairach coordinates				
Region	Laterality	x	y	z	Size	t-score*
**Frontal Lobe**		**Experimental>Control**				
Superior frontal gyrus (BA 6)	L	−1	4	56	35	2.9
Ventrolateral prefrontal cortex (BA 46)	R	53	29	16	27	2.6
Ventromedial prefrontal cortex (BA 10)	R	9	53	−4	99	3.3
**Temporal Lobe**		**Experimental>Control**				
Middle temporal gyrus (BA 21)	L	−63	−5	−6	20	3.1
Middle temporal gyrus (BA 21)	R	65	−19	−14	28	3.1
**Limbic Lobe**		**Experimental>Control**				
Amygdala	L	−17	−7	−16	34	3.1
Parahippocampal gyrus (BA 36)	L	−27	−44	−6	121	3.0
Pregenual anterior cingulate cortex (BA 24)	L	−5	37	2	108	3.0
Amygdala	R	15	−7	−18	32	3.1
Parahippocampal gyrus (BA 36)	R	25	−43	−8	21	3.2
Periamygdaloid cortex	R	29	3	−20	40	3.3
Posterior cingulate cortex (BA 30)	R	17	−47	4	124	3.1
Posterior cingulate cortex (BA 30)	R	2	−53	5	123	2.8
**Frontal Lobe**		**Control>Experimental**				
Ventrolateral prefrontal cortex (BA 44)	L	−55	9	22	31	−2.8
Middle frontal gyrus (BA 8)	R	37	17	48	62	−3.3

Differences in regional BOLD activity between the experimental and control (sham) groups associated with the Happy Memories condition (versus Rest) for the Transfer run.

BA – Brodmann areas; L – left; R – right;

*p<0.05, uncorrected (Size – cluster size, minimum 20 voxels).

### Functional Connectivity Analysis

**Figure 8:**
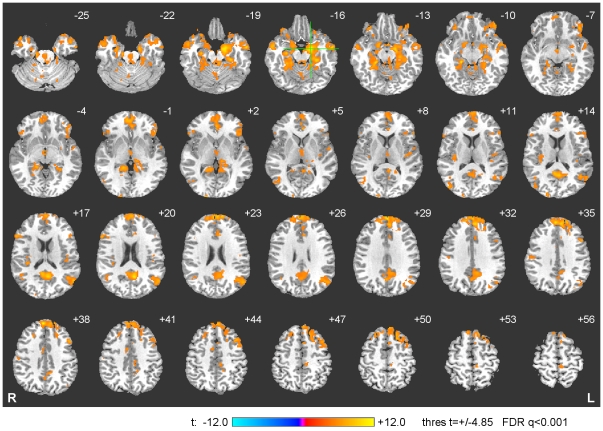
Functional Connectivity Analysis for the Amygdala Network. The group functional connectivity analysis using a seed ROI in the left amygdala region revealed a fronto-temporo-limbic network (see text for details and Table 3 for coordinates). The connectivity maps are projected on a representative single-subject T1 template in the Talairach space. The coordinates and orientation of each slice are described in the legend for Figure 7. The green crosshairs mark the center of the seed ROI for the connectivity analysis.

Results of group functional connectivity analysis for the Transfer run within the experimental group are shown in Figure 8. The center of the seed ROI for this analysis was chosen at (−17, −7, −16), i.e. the point within the left amygdala region exhibiting the maximum training effect according to Table 2. The seed ROI was spherical with 5 mm radius. Properties of the connectivity centers in Figure 8 are specified in Table 3. The connectivity centers were identified using the cluster approach with threshold *q*<0.001 and the minimum cluster size of 20 voxels. The functional connectivity pattern for the left amygdala reveals a fronto-temporo-limbic network, including bilateral SFG, bilateral VLPFC, bilateral MFPC, bilateral DMPFC, bilateral lateral orbitofrontal cortex (LOFC), left middle frontal gyrus (MidFG), and right VMPFC in the frontal lobe; bilateral MTG in the temporal lobe; and bilateral amygdala, bilateral PHG, bilateral PCC, left pregenual ACC, right subgenual ACC, and right HC in the limbic lobe. The connectivity pattern also includes thalamus and bilateral insula. For several regions in Figure 8, locations of the connectivity maxima (Table 3) are spatially close to the corresponding connectivity peak locations reported in a recent meta-analytic study of the amygdala functional connectivity [49]. These regions include bilateral amygdala, left LOFC (BA 47), left pregenual ACC (BA 24), left PCC (BA 31), left MTG (BA 39), left SFG (BA 9).

**Table 3:** Functional Connectivity Analysis of the Amygdala Network.

		Talairach coordinates			
Region	Laterality	x	y	z	t-score*
**Frontal Lobe**					
Ventrolateral prefrontal cortex (BA 45)	L	−51	25	10	7.2
Lateral orbitofrontal cortex (BA 47)	L	−41	31	−6	8.5
Middle frontal gyrus (BA 8)	L	−39	15	45	9.3
Superior frontal gyrus (BA 6)**	L	−9	17	62	8.7
Medial frontal polar cortex (BA 9)	L	−1	55	29	11.0
Dorsomedial prefrontal cortex (BA 9)**	L	−6	45	34	10.3
Ventrolateral prefrontal cortex (BA 45)	R	55	21	18	8.1
Lateral orbitofrontal cortex (BA 47)	R	43	25	−2	6.5
Superior frontal gyrus (BA 8)**	R	9	31	54	7.0
Dorsomedial prefrontal cortex (BA 9)**	R	3	47	38	11.7
Medial frontal polar cortex (BA 10)**	R	5	56	21	9.2
Ventromedial prefrontal cortex (BA 10)	R	10	49	−3	9.6
**Temporal Lobe**					
Middle temporal gyrus (BA 39)	L	−53	−63	18	8.3
Middle temporal gyrus (BA 21)	L	−57	−5	−18	9.5
Middle temporal gyrus (BA 21)	R	55	−3	−14	10.4
**Limbic Lobe**					
Amygdala	L	−17	−7	−14	11.5
Parahippocampal gyrus (BA 36)	L	−29	−29	−17	10.0
Pregenual anterior cingulate cortex (BA 24)**	L	−3	34	5	8.2
Posterior cingulate cortex (BA 31)	L	−7	−57	20	10.9
Amygdala	R	15	−1	−14	8.4
Parahippocampal gyrus (BA 36)	R	21	−33	−10	10.8
Hippocampus	R	29	−15	−17	8.7
Parahippocampal gyrus (BA 35)	R	15	−41	−2	10.0
Subgenual anterior cingulate cortex (BA 25)	R	1	11	−8	6.7
Posterior cingulate cortex (BA 30)	R	5	−51	16	9.9
**Sub-lobar Regions**					
Thalamus	L	−11	−29	0	6.9
Posterior insula	L	−45	−15	10	6.4
Posterior insula	R	33	−31	18	6.8

Functional connectivity results for the Transfer run using a seed ROI in the left amygdala region.

BA – Brodmann areas; L – left; R – right.

*q(FDR)<0.001 (minimum 20 voxels).

**Functional connectivity with left amygdala increases across the neurofeedback and Transfer runs.

**Figure 9:**
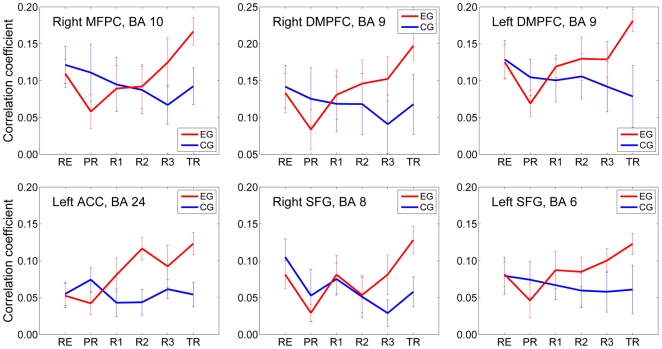
Enhancement in Functional Connectivity with the Left Amygdala during the Neurofeedback Training. For the subjects in the experimental group (EG, red), the functional connectivity with the left amygdala increased across the neurofeedback training runs (PR, R1, R2, R3) and the Transfer run (TR) for the right medial frontal polar cortex (MFPC, BA 10), bilateral dorsomedial prefrontal cortex (DMPFC, BA 9), left pregenual anterior cingulate cortex (ACC, BA 24), and bilateral superior frontal gyrus (SFG, BA 6,8). In contrast, no significant connectivity changes were observed for the same regions for the Control (sham) group (CG, blue).

To explore changes in functional connectivity within the amygdala network, one-way ANOVA trend analyses on correlation coefficient values across five runs (PR, R1, R2, R3, TR) were performed (see Data Processing and Analysis above). For the experimental group, six regions within the amygdala network demonstrated a *significant* increase in functional connectivity over the course of the neurofeedback training. The average correlation coefficient values (±s.e.m.) for the corresponding ROIs are exhibited in Figure 9 (EG, red), and the ROI centers are marked by ** in Table 3. These regions include: (1) right medial frontal polar cortex (*F*(4,52) = 3.95, *P*<0.007; Linear: *F*(1,13) = 22.0, *P*<0.0004; Quadratic: *F*(1,13) = 0.26, *P*<0.622; Cubic: *F*(1,13) = 0.33, *P*<0.576); (2) right dorsomedial prefrontal cortex (*F*(4,52) = 5.10, *P*<0.002; Linear: *F*(1,13) = 22.0, *P*<0.0004; Quadratic: *F*(1,13) = 0.02, *P*<0.883; Cubic: *F*(1,13) = 2.41, *P*<0.145); (3) left dorsomedial prefrontal cortex (*F*(4,52) = 5.46, *P*<0.001; Linear: *F*(1,13) = 28.0, *P*<0.0001; Quadratic: *F*(1,13) = 0.007, *P*<0.934; Cubic: *F*(1,13) = 9.03, *P*<0.010); (4) left pregenual anterior cingulate cortex (*F*(4,52) = 5.44, *P*<0.001; Linear: *F*(1,13) = 14.0, *P*<0.002; Quadratic: *F*(1,13) = 2.85, *P*<0.115; Cubic: *F*(1,13) = 3.03, *P*<0.105); (5) right superior frontal gyrus (*F*(4,52) = 4.58, *P*<0.003; Linear: *F*(1,13) = 14.5, *P*<0.002; Quadratic: *F*(1,13) = 0.56, *P*<0.467; Cubic: *F*(1,13) = 6.07, *P*<0.028); (6) left superior frontal gyrus (*F*(4,52) = 3.06, *P*<0.025; Linear: *F*(1,13) = 7.63, *P*<0.016; Quadratic: *F*(1,13) = 0.09, *P*<0.767; Cubic: *F*(1,13) = 1.30, *P*<0.275). Note that the overall within-subject connectivity enhancement effect and the linear trend effect for these regions are significant across all experimental runs (i.e. with the Rest run included in the trend analysis), as demonstrated by the following statistics for the same six regions, respectively: (1) *F*(5,65) = 3.34, *P*<0.01 (Linear: *F*(1,13) = 10.48, *P*<0.006); (2) *F*(5,65) = 3.53, *P*<0.007 (Linear: *F*(1,13) = 11.12, *P*<0.005); (3) *F*(5,65) = 4.15, *P*<0.002 (Linear: *F*(1,13) = 13.06, *P*<0.003); (4) *F*(5,65) = 5.20, *P*<0.0004 (Linear: *F*(1,13) = 21.73, *P*<0.0004); (5) *F*(5,65) = 3.69, *P*<0.005 (Linear: *F*(1,13) = 4.68, *P*<0.050); (6) *F*(5,65) = 2.64, *P*<0.031 (Linear: *F*(1,13) = 6.37, *P*<0.025). It should be noted also that all six regions, demonstrating significant connectivity enhancement with the left amygdala are located near the brain's medial plane (Table 3).

To examine whether functional connectivity with the left amygdala region was affected by sham neurofeedback, the same statistical analyses were performed for the control group using the same ROIs to determine average correlation coefficient values. The results are exhibited in Figure 9 (CG, blue), and are characterized by the following statistics: (1) right medial frontal polar cortex: *F*(4,52) = 0.33, *P*<0.855 (Linear: *F*(1,13) = 0.19, *P*<0.671); (2) right dorsomedial prefrontal cortex: *F*(4,52) = 0.38, *P*<0.854 (Linear: *F*(1,13) = 0.28, *P*<0.607); (3) left dorsomedial prefrontal cortex: *F*(4,52) = 0.23, *P*<0.921 (Linear: *F*(1,13) = 0.67, *P*<0.428); (4) left pregenual anterior cingulate cortex: *F*(4,52) = 0.85, *P*<0.502 (Linear: *F*(1,13) = 0.29, *P*<0.598); (5) right superior frontal gyrus: *F*(4,52) = 0.42, *P*<0.792 (Linear: *F*(1,13) = 0.006, *P*<0.937); (6) left superior frontal gyrus: *F*(4,52) = 0.09, *P*<0.984 (Linear: *F*(1,13) = 0.13, *P*<0.725). Therefore, no significant changes in functional connectivity with the left amygdala were observed for the same six regions for the control (sham) group.

## Discussion

In this study, we investigated the feasibility of training healthy volunteers to regulate the level of activation in their left amygdala using rtfMRI neurofeedback. The results of this study demonstrated that, given appropriate direction, practice, and rtfMRI neurofeedback information, individuals learned to significantly enhance the regional BOLD activity in the amygdala by contemplating positive autobiographical memories within a short training session. The BOLD fMRI signal in the left amygdala increased with the number of neurofeedback runs, resembling a progressive learning effect reported by previous studies for other brain regions: the right anterior insula [18], somatomotor cortex [7], anterior cingulate cortex [12], inferior frontal gyrus [16], parahippocampal cortex [6]. Moreover, the study confirmed that rtfMRI neurofeedback training affords an effective noninvasive approach for modulating regional brain activity. These results thus hold potential clinical relevance for studies involving psychiatric conditions that are tractable by cognitive-behavioral approaches, such as major depressive disorder, obsessive-compulsive disorder, and anxiety disorders.

The amygdala plays major roles in the neural processing underlying emotional behavior, and plays crucial roles in evaluating the salience of experiential stimuli, aversive and appetitive conditioning [23], [50], contextual conditioning [51], enhancement of memory formation by emotional or arousing stimuli [52], and social dominance hierarchy processing [53], [54]. Amygdala activity changes during the modulation of emotional experience [55], [56]. Abnormalities of amygdala function are reported in a plethora of psychiatric disorders [57]–[59] and in individuals with genetic risk factors for such disorders [60], [61]. Hence, the volitional modulation of amygdala activity using rtfMRI neurofeedback training might be relevant for the development of novel therapeutic approaches to psychiatric disorders [62].

Although previous rtfMRI neurofeedback training studies investigated the possibility of training healthy humans to control amygdala activity during self-induced sadness [17], [19], these studies did not include a sham condition and only assessed potential learning effects. In our study, we addressed these issues by employing a short-term rtfMRI neurofeedback protocol that focused on the training of the left amygdala, but addressed the specificity of this effect by including sham neurofeedback from another region. The results demonstrated that individuals were able, by contemplating positive autobiographical memories, to increase the level of fMRI activation in the left amygdala through neurofeedback training.

Recent evidence from quantitative meta-analyses of functional neuroimaging studies has suggested a functional dissociation between left and right amygdala in terms of temporal dynamics [20], [26], [27], with the left amygdala involved in more detailed and elaborate stimulus evaluation and the right amygdala involved in rapid, short and relatively automatic detection of emotional stimuli. Therefore, the increase in the left amygdala activity during neurofeedback training in our study may reflect ongoing processing of emotionally salient memories. Consistent with this hypothesis, predominantly left-sided amygdala activation has been hypothesized to relate to left-lateralized higher cognitive processes associated with recognition and analytic processing [22] and to cognitive representation of emotion [29]. Other studies reported that hemodynamic responses of the amygdala are more prominent on the *left* side in response to positively valenced stimuli, such as to happy face stimuli presented below the level of conscious awareness [63].

The enhanced control over left amygdala BOLD activity appeared to specifically result from rtfMRI-induced learning. The control group underwent the same rtfMRI neurofeedback procedure as the experimental group, but received sham neurofeedback information corresponding to BOLD activity in the left HIPS, a region that has been consistently implicated in numeric processing [38]. Although the control group initially showed a similar level of BOLD activity in the target ROIs as the experimental group, the control group did not differentially modulate activity in either the HIPS or the amygdala across runs. Therefore, the observed learning effect appeared attributable to ROI-specific neurofeedback training rather than to nonspecific aspects of task performance such as repetition or practice effects.

The observed training effect generalized to the Transfer run, in which neurofeedback was no longer provided. During this run, the participants were instructed to contemplate positive autobiographical memories in the absence of rtfMRI neurofeedback. The results suggested that the subjects continued to use the learning acquired during the preceding neurofeedback trials. A similar transfer effect was recently reported for the anterior insula during rtfMRI neurofeedback training [18]. Moreover, the training effect was associated with the participants' insight into their own emotional experience. The results indicate that the better the individuals were at identifying (but not describing) their emotional experience, the better they performed at regulating their left amygdala activity across the training (Fig. 5A). This finding supports previous studies implicating amygdala involvement in self-induced mood states and reported correlations between amygdala activity and emotional experience [22], [30], [64]. Additionally training effect was associated with the susceptibility to anger (Fig. 5B), suggesting that individuals' performance during neurofeedback training may be inversely correlated with their sensitivity to other people's negative emotions, particularly with their susceptibility to anger.

Although the right amygdala showed a nominal increase in BOLD activity across the neurofeedback trials, this effect was only marginally significant. The statistically significant self-regulation was only specific to the target ROI—the left amygdala. These data are compatible with those from studies in psychiatric disorders which showed a lack of correlation between the left and right amygdala metabolism [65]. Similarly, rtfMRI neurofeedback training anatomically specific to the right anterior insula was reported recently [18].

Analysis of the participants' cardiac and respiratory waveforms, recorded simultaneously with fMRI for each run, revealed no statistically significant differences between the experimental and control groups in terms of their cardiac and respiration rates for any of the runs. Additionally, no correlations were found between the left amygdala BOLD activations and the subjects' cardiac and respiratory rates. These results suggest that the pronounced differences in the left amygdala activation levels across Runs 1–3 between the two groups cannot be attributed to differences in cardiorespiratory effects observed using the standard physiological recordings and simple physiological rate analyses. It should be noted also that the largest relative variations in both cardiac and respiratory rates between the Happy Memories and Rest conditions occurred during Run 1, i.e. close to the middle of the neurofeedback experiment.

The whole-brain voxel-wise analyses showed that the training to modulate left amygdala activity while contemplating positive autobiographical memories engaged a fronto-temporo-limbic network that is implicated in emotion processing and autobiographical memory retrieval [20], [66]. The lateral orbitofrontal cortex, the ventrolateral PFC, and the medial portions of FPC have been implicated in prefrontal cortical systems that share extensive anatomical connections and engage in functional interactions with the amygdala during emotional learning and behavior [67]–[69]. The network engaged in autobiographical memory involves the hippocampus, parahippocampal gyrus, ventrolateral PFC, medial PFC, ACC, PCC, and temporoparietal junction [70], which showed changes in BOLD activity during the Happy Memories task (Table 1). These regions exhibited a significant experimental versus control group contrast for the Happy Memories (versus Rest) condition (Table 2).

Analysis of functional connectivity of the amygdala network revealed connectivity with fronto-temporo-limbic network (frontal: VLPFC, DMPFC, MFPC, LOFC, SFG, MidFG; temporal: MTG; limbic: PHG, HC, ACC, PCC), as well as sub-lobar structures such as the thalamus and insula. These findings are consistent with a previous meta-analytic connectivity modeling analysis that investigated the functional connectivity of the amygdala based on human neuroimaging studies and anatomical studies in nonhuman primates [49]. Six regions within the network – right MFPC, left and right DMPFC, left pregenual ACC, left and right SFG – demonstrated a significant increase in functional connectivity with the left amygdala over the course of neurofeedback training (Fig. 9 and Table 3). These findings are compatible with the reciprocal connections extant between the amygdala and the ACC, MPFC, and MFPC, through which amygdala activity is modulated by activity within the PFC, allowing the modulation of emotional processes by higher cognitive processes, such as autobiographical memory [62], [69]. Previous neuroimaging studies have demonstrated functional roles for these projections during emotional regulation, as studied, for example, within the context of applying cognitive strategies such as reappraisal to alter functional activity in the amygdala during the processing of emotional stimuli [56], [71].

Some limitations of our study design merit comment. First, the participants' actual emotional experience was not independently assessed in the present study. Thus we cannot comment on whether their mood state became more positive while contemplating positive autobiographical memories. Second, individual performance during neurofeedback training depended on the subjects' ability to alter left amygdala activity by contemplating positive autobiographical memories while receiving neurofeedback information. This led to a relatively large inter-subject variability of the results. For the participants in the experimental group, the average left amygdala activation for all Happy Memories conditions across Runs 1–3 varied from +0.60% (the best performance) to −0.13% (the worst performance). While the differences in the subjects' ability to identify feelings partly accounted for this variability (Fig. 5A), other individual factors (e.g., learning ability, attention, focus, motivation) likely contributed as well. Repeating the rtfMRI neurofeedback session multiple times can be expected to improve the efficiency of neurofeedback training for most participants. It is conceivable that adding new training sessions across multiple days may further increase the training effect and enhance the subjects' skills at modulating amygdala activity using neurofeedback. Other means of enhancing neurofeedback training may involve the development of adaptive training paradigms, in which the target activation level (set by the blue bar in Fig. 1) is adjusted, either in real time or between runs, based upon individual performance. Finally, more efficient methods, such as the induction of emotion using emotionally valenced stimuli, may conceivably be identified, which would benefit neurofeedback training of the amygdala and other regions involved in emotion processing. In terms of the experimental protocol optimization, addition of a final resting run would allow a direct comparison of resting-state functional connectivity networks before and after the neurofeedback training, as shown recently in [72].

In summary, our findings demonstrate that healthy, neurofeedback-naive subjects can learn to regulate their amygdala activation using positive autobiographical memory retrieval while receiving rtfMRI neurofeedback. In contrast to the sham feedback from the HIPS region, the feedback provided from the left amygdala resulted in a significant monotonic BOLD signal increase during rtfMRI neurofeedback training, and this effect persisted during the Transfer run, in which no feedback was provided. Across the individual subjects from the experimental group, the training effect in the LA BOLD activity correlated inversely with scores on the Difficulty Identifying Feelings subscale of the Toronto Alexithymia Scale, suggesting that the better subjects rated their ability to identify their emotions, the more effectively they learned to regulate LA activity via training. Furthermore, the whole brain data analysis revealed significant group differences (experimental versus control) for the Happy Memories versus Rest condition. The comparison of the Happy Memories and Count conditions revealed significant Happy Memories>Count contrast in a fronto-temporo-limbic network, and significant Count>Happy contrast in a parietal network. Functional connectivity analysis of the amygdala network demonstrated significant widespread correlations among regional BOLD signal changes in a fronto-temporo-limbic network. Additionally, we identified six regions – right MFPC, bilateral DMPFC, left ACC, and bilateral SFG – where the functional connectivity with the left amygdala increased across the rtfMRI neurofeedback runs and the Transfer run.

Further studies are needed to determine whether the findings provided in this proof-of-concept study have the potential to significantly advance our understanding of the pathophysiology of neuropsychiatric disorders. For example, the modulation of amygdala activity using rtfMRI neurofeedback training may be particularly relevant for the development of novel approaches for optimizing cognitive-behavioral therapeutic interventions in post-traumatic stress disorder (PTSD) or major depressive disorders. Because exaggerated hemodynamic responses of the amygdala to fearful faces or traumatic reminders are consistent pathological constructs in PTSD [73], [74], investigating whether patients are able to down-regulate their amygdala activity through learned modulation and whether such learning may lead to behavioral changes would be a important clinical target for future research. Moreover, because of the importance in prefrontal cortical modulation of amygdala activity during emotional processing, the functional connectivity between brain areas (e.g., MPFC and amygdala) could be used as specific physiological outcome parameters in future rtfMRI neurofeedback training studies.
